# Varenicline and risk of psychiatric conditions, suicidal behaviour, criminal offending, and transport accidents and offences: population based cohort study

**DOI:** 10.1136/bmj.h2388

**Published:** 2015-06-03

**Authors:** Yasmina Molero, Paul Lichtenstein, Johan Zetterqvist, Clara Hellner Gumpert, Seena Fazel

**Affiliations:** 1 Department of Clinical Neuroscience, Center for Psychiatry Research, Karolinska Institutet, 171 76 Stockholm, Sweden; 2 Department of Medical Epidemiology and Biostatistics, Karolinska Institutet, Box 281, 171 77 Stockholm; 3 Department of Psychiatry, University of Oxford, Warneford Hospital, Oxford OX3 7JX, UK

## Abstract

**Objective** To examine associations between varenicline and the incidence of a range of adverse outcomes.

**Design** Population based cohort study using within person analyses to control for confounding by indication.

**Setting** Whole population of Sweden.

**Participants** 7 917 436 people aged 15 and over, of whom 69 757 were treated with varenicline between 2006 and 2009.

**Main outcome measures** Incidence of new psychiatric conditions, suicidal behaviour, suspected and convicted criminal offending, transport accidents, and suspected and convicted traffic offences.

**Results** In the whole population, 337 393 new psychiatric conditions were diagnosed during follow-up. In addition, 507 823 suspected and 338 608 convicted crimes, 40 595 suicidal events, 124 445 transport accidents, and 99 895 suspected and 57 068 convicted traffic crimes were recorded. Within person analyses showed that varenicline was not associated with significant hazards of suicidal behaviour, criminal offending, transport accidents, traffic offences, or psychoses. However, varenicline was associated with a small increase in the risk of anxiety conditions (hazard ratio 1.23, 95% confidence interval 1.01 to 1.51) and mood conditions (1.31, 1.06 to 1.63), which was only seen in people with pre-existing psychiatric disorders.

**Conclusions** Concerns that varenicline is associated with an increased risk of many adverse outcomes, including suicidality and accidents, are not supported in this observational study. The small increase in risk of two psychiatric conditions in people with pre-existing psychiatric disorders needs to be confirmed using other research designs.

## Introduction

Around 1.3 billion people in the world smoke tobacco,^1^ and tobacco use is the second leading risk factor contributing to global disease burden,^2^ accounting for 9% of deaths globally and 18% of deaths in high income countries.^3^ Smoking cessation treatments include nicotine replacement therapies and nicotine-free prescription drugs. Increasingly, tobacco dependence is treated with varenicline (marketed as Champix or Chantix). Varenicline acts as a partial agonist of the nicotinic acetylcholine receptor, simultaneously relieving withdrawal symptoms and decreasing rewards from smoking. Multiple studies have shown that varenicline is more efficacious than placebo, bupropion, or single forms of nicotine replacement therapy.^4^ Between approval by the Food and Drug Administration in 2006 and mid-2011, 8.9 million people were treated with varenicline in the United States.^5^ In the United Kingdom, varenicline was prescribed to more than 800 000 patients in primary care in 2009 and is one of the most commonly used smoking cessation drugs.^6^

After varenicline’s introduction on the market, reports of suicidality and depression emerged in post-marketing surveillance and eventually led to warnings issued by regulatory agencies in Europe and a black box warning in the United States.^7^ ^8^ Furthermore, varenicline has been reported to increase the risk of traffic accidents,^9^ and it has been restricted or prohibited in several transportation industry professions, including pilots, air traffic controllers, truck and bus drivers, and certain military personnel.^9^ ^10^ Some weaker evidence also suggests an increased risk of violence and psychosis.^10^ ^11^ ^12^ ^13^ ^14^ ^15^ However, these increased risks are based on post-marketing surveillance and case reports,^9^ ^11^ ^12^ ^13^ ^14^ ^15^ ^16^ ^17^ ^18^ ^19^ which are not consistent with observational data and randomised controlled trials that have found no association between varenicline and depression, suicidality, or violence.^4^ ^20^ ^21^ ^22^ ^23^ ^24^ ^25^ ^26^ ^27^ ^28^ These inconsistencies could be explained by differences in study designs, confounding by comorbid psychiatric disorders or by indication bias (that is, the same factors may influence both institution of treatment and outcomes), or reporting bias.^29^ Moreover, people with mental health problems make up a substantial proportion of smokers.^30^ Although no safety concerns have been raised in randomised controlled trials of varenicline in people with bipolar disorder, major depression, and schizophrenia, trials have comprised small samples,^31^ ^32^ resulting in limited statistical power to detect rare events.^33^ ^34^ ^35^ ^36^ ^37^

To overcome limitations of previous research, we used a within person design in which measurements are made repeatedly over time, and treatment and non-treatment periods are compared within the same person. Use of this approach, in which each person serves as his or her own control, thus adjusts for all time invariant confounders during follow-up (genetic factors, all factors up to the start of follow-up, and those that remain constant during follow-up). Through this design, selection effects can be minimised, unmeasured confounders can be adjusted for, and confounding by indication can be overcome.^29^

We report a within person design to examine the association between varenicline and incidence of new psychiatric conditions, suicidal behaviour, suspected and convicted crimes, transport accidents, and suspected and convicted traffic offences in a large population based Swedish cohort followed from 2006 to 2009.

## Methods

In the total population of Sweden aged 15 and over (n=7 917 436), we identified 69 757 people who had varenicline prescribed between 22 November 2006 (that is, the introduction of varenicline in Sweden) and 31 December 2009. We collected information on individuals from Swedish population based registers with national coverage, and registers were linked using each person’s unique identification number.

### Varenicline treatment

The Prescribed Drug Register includes information on all prescribed and collected medical drugs since July 2005.^38^ We defined varenicline treatment as at least one collected prescription of varenicline (N07BA03) between 22 November 2006 and 31 December 2009. Varenicline is recommended to be taken as a 12 week treatment,^39^ so we defined a treatment period as starting at the date of the first collected prescription and ending 12 weeks later. Because varenicline is often divided into several prescriptions for the same 12 week treatment, we considered collected prescriptions within 12 weeks of the first collected prescription to be part of the same treatment period. We considered prescription collections occurring more than 12 weeks after a previous collection to be a new treatment period, starting at the date of the next collected prescription.

### Outcomes

#### Crimes

We defined crimes as all offences in the penal code, except traffic offences. We extracted information on convicted crimes for people aged 15 and older (the age of criminal responsibility) from the National Crime Register, including all convictions in Swedish district courts.^40^ We extracted suspected crimes from the Register of People Suspected of Offences and included all people suspected of crime after a completed investigation by police, the customs authority, or the prosecution service.^41^

#### Incidence of new psychiatric conditions

Information on incidence of new psychiatric conditions came from the Patient Register,^42^ which includes diagnoses from both hospital admissions and outpatient visits in specialised care. Diagnoses received during planned visits (that is, follow-ups and referrals) were excluded from the analyses. Although this gives a more conservative estimate of psychiatric conditions, we used this measure to avoid overestimation of diagnoses; the diagnosis that is the reason for starting treatment is also coded during follow-ups and referrals, regardless of current symptoms. We also did sensitivity analyses including planned visits. Psychiatric conditions included three diagnostic categories: psychoses ( ICD-10 (international classification of diseases, 10th revision) codes: F20-F29), mood conditions (F30-F39), and anxiety conditions (F40-F45, F48).

#### Suicidal behaviour

We defined suicide attempts and suicides as emergency inpatient or outpatient hospital visits or death due to intentional self harm (ICD-10: X60-X84). We collected information on suicide attempts from the Patient Register and information on suicides from the Cause of Death Register.^43^

#### Transport accidents and traffic offences

We defined transport accidents as an emergency inpatient or outpatient hospital visit or death due to transport accidents (ICD-10: V00-V99). We defined traffic offences as convictions or suspicions of traffic offences (defined as crimes against the road traffic offences act and including reckless driving, unlawful driving, hit and run offences, causing death or injury by driving, and moving violations). Information on transport accidents came from the Patient Register and the Cause of Death Register. Information on convicted traffic offences came from the National Crime Register and information on suspected traffic offences from the Register of People Suspected of Offences.

#### Substance abuse

We collected information on alcohol use disorders and dependence (ICD-9: 291, 303, 305A, 980; ICD-10: F10), drug misuse and dependence (ICD-9: 292, 304, 977W, 977X; ICD-10: F11–F16, F18–F19), and nicotine dependence (ICD-9: 305B; ICD-10: F17) from the Patient Register.

### Patient involvement

There was no patient involvement in this study.

### Statistical analyses

We followed people from 22 November 2006 to 31 December 2009. A between person Cox proportional hazards regression analysis compared average rates of each outcome during varenicline treatment for all people with rates during non-treatment for all people. In this analysis, we split follow-up into the period before the first outcome, periods between outcomes, and the period after the last outcome. We measured time at risk from the start of each period and used treatment as a time varying covariate. We calculated robust standard errors to account for correlations between periods within the same person. Analyses were adjusted for sex and age in a second step.

The principal analyses were within person stratified Cox proportional hazards regression, with each person entering as a separate stratum in the analysis and serving as his/her own control. The obtained hazard ratio is thus adjusted for (that is, stratified on) all potential time invariant confounders within each person. To adjust for age, which is a time varying potential confounder, we added age to the model as a time varying covariate, with one factor for each whole year. We adjusted periods of treatment and non-treatment for migration, imprisonment, institutional youth care, hospital admission, and death. We identified migrations and deaths by linking people to the Migration and Cause of Death Registers. We accounted for periods in prison and institutional youth care by linkage to the Prison Register and estimated periods in hospital by using the Patient Register. In the within person stratified Cox proportional hazards regression, only people who change treatment status contribute directly to the estimate. All other people contribute indirectly through the estimates of other covariates. As the covariates in the within person stratified Cox proportional hazards regression are time varying, we did not test for the proportional hazards assumption.

More information on this approach is provided in studies of drugs for attention-deficit/hyperactivity disorder, antipsychotics, and mood stabilisers.^44^ ^45^ ^46^ ^47^ We used SAS version 9.4 for all analyses.

### Sensitivity analyses

In sensitivity analyses, we analysed separately each of the three diagnostic categories included in the definition of psychiatric conditions (mood conditions, anxiety conditions, and psychoses). Firstly, we included everyone in the cohort in the analyses. Secondly, to test for confounding by pre-existing psychiatric disorders, we included only those with pre-existing psychiatric diagnoses (ICD-9: 295-302, 307-316; ICD-10: F20-F48, F50-F69, F90-F98; diagnosed before 1 November 2006). Thirdly, we included only people without prior psychiatric disorders in the analyses. Furthermore, we did sensitivity analyses including both emergency and planned inpatient and outpatient visits for incidence of new psychiatric conditions.

In further sensitivity analyses, we examined whether the increased risk of psychiatric conditions could be the result of nicotine withdrawal syndrome, which is a potential time varying confounder. We used a comparison group with nicotine dependence—people who had collected at least one prescription for the smoking cessation drug bupropion (N06AX12) during follow-up (n=63 265). In this sensitivity analysis, we included only people who had been treated with either varenicline or bupropion; those who had been treated with both varenicline and bupropion during follow-up (n=11 386) were excluded. We then did a between person Cox proportional hazards regression, comparing average rates of mood and anxiety conditions during varenicline treatment with rates during non-treatment.

## Results

Between 22 November 2006 and 31 December 2009, 43 861 women and 25 896 men were treated with varenicline in Sweden (see table 1 for background characteristics). During this time period, 5.4% in the varenicline population were suspected of a crime and 4.6% were diagnosed as having a new psychiatric condition; the rate of serious traffic related incidents was 1.4% and 0.9% received medical care for suicidal behaviours in the varenicline population. In the same time period, 4.0% in the non-treated population were suspected of a crime, 2.2% were diagnosed as having a new psychiatric condition, 1.4% received medical care for transport related accidents, and 0.3% received medical care for suicidal behaviours.

**Table 1 tbl1:** Descriptive data for varenicline cohort and non-treated cohort in Sweden, 2006-09. Values are numbers (percentages)

	Varenicline cohort (n=69 757)	Non-treated cohort* (n=7 847 679)
**Characteristics at baseline 2006**		
Women	43 861 (62.9)	3 964 263 (50.5)
Men	25 896 (37.1)	3 883 417 (49.5)
Age distribution:		
<20	408 (0.6)	982 116 (12.5)
20-29	3744 (5.4)	1 084 733 (13.8)
30-39	9226 (13.2)	1 222 610 (15.6)
40-49	17 375 (24.9)	1 221 021 (15.6)
50-59	21 480 (30.8)	1 170 086 (14.9)
60-69	14 447 (20.7)	1 022 055 (13.0)
≥70	3077 (4.4)	1 143 656 (14.6)
Psychiatric diagnoses:		
Pre-existing psychiatric diagnosis†	9391 (13.5)	484 536 (6.2)
Lifetime alcohol misuse diagnosis‡	5562 (8.0)	197 988 (2.5)
Lifetime drug misuse diagnosis‡	2633 (3.8)	80 535 (1.0)
Lifetime nicotine dependence diagnosis‡	2379 (3.4)	19 392 (0.3)
**Characteristics during follow-up (22 November 2006 to 31 December 2009)**		
Inpatient or outpatient care:		
New psychiatric conditions	3213 (4.6)	168 869 (2.2)
Anxiety conditions	1816 (2.6)	88 905 (1.1)
Mood conditions	1717 (2.5)	84 931 (1.1)
Psychoses	320 (0.5)	24 384 (0.3)
Suicidal behaviour	657 (0.9)	26 093 (0.3)
Crimes:		
Convicted of any crime	2256 (3.2)	204 508 (2.6)
Suspected of any crime	3782 (5.4)	311 914 (4.0)
Transport accidents and traffic offences:		
Transport accident	989 (1.4)	108 612 (1.4)
Convicted of traffic offence	328 (0.5)	36 271 (0.5)
Suspected of traffic offence	440 (0.6)	46 572 (0.6)

*All people in cohort who were not treated with varenicline during follow-up.

†Diagnosed before 1 November 2006.

‡Diagnosed between 1 January 1987 and 31 December 2009.

Our unadjusted, between person Cox proportional hazards regression showed that people with varenicline prescriptions had significantly higher hazards of a range of adverse outcomes compared with people who were not treated with varenicline. These included the incidence of new psychiatric conditions (hazard ratio 3.29, 95% confidence interval 2.99 to 3.63), of suicidal behaviour (3.44, 2.64 to 4.47), of being suspected of a crime (1.45, 1.30 to 1.62), and of being convicted of a crime (1.18, 1.05 to 1.32). Varenicline prescription was, however, not associated with significantly increased hazards of transport accidents or traffic offences. When we adjusted for age and sex in the between person Cox proportional hazards regression, people with varenicline prescriptions had significantly increased hazards of all seven outcomes compared with non-treated people (table 2).

**Table 2 tbl2:** Associations between varenicline and adverse outcomes from unadjusted and progressively more adjusted analyses

Outcome	No of events at within person level/No of events at between person level	Hazard ratio (95% CI)		
		Between person, unadjusted	Between person, adjusted for sex and age	Within person*
Incidence of new psychiatric conditions	6910/337 393	3.29 (2.99 to 3.63)	2.78 (2.63 to 2.93)	1.18 (1.05 to 1.31)
Suicidal behaviour	1077/40 595	3.44 (2.64 to 4.47)	4.06 (3.12 to 5.28)	1.00 (0.72 to 1.37)
Suspected of any crime	6873/507 823	1.45 (1.30 to 1.62)	2.33 (2.08 to 2.60)	1.10 (0.97 to 1.24)
Convicted of any crime	3252/338 608	1.18 (1.05 to 1.32)	1.88 (1.68 to 2.11)	0.96 (0.79 to 1.16)
Transport accidents	1129/124 445	1.05 (0.87 to 1.28)	1.46 (1.20 to 1.78)	1.01 (0.69 to 1.47)
Suspected of traffic offence	772/99 895	1.17 (0.88 to 1.55)	1.74 (1.31 to 2.32)	1.24 (0.84 to 1.84)
Convicted of traffic offence	483/57 068	1.13 (0.84 to 1.52)	1.81 (1.34 to 2.44)	1.30 (0.77 to 2.20)

*Within person model compares rate of adverse events when person is prescribed varenicline with rate when same person is not prescribed varenicline.

To account for residual confounders that might explain the observed increased hazards, we then compared the rates of each outcome within the same person (that is, with each person serving as his or her own control), using the within person design. This showed that being treated with varenicline was not associated with significantly increased hazards of suspected or convicted crimes, suicidal behaviour, transport accidents, or suspected or convicted traffic offences (table 2). However, varenicline was associated with an increased hazard of new psychiatric conditions (hazard ratio 1.18, 1.05 to 1.31). To further examine the associations between varenicline and incidence of new psychiatric conditions, we analysed each diagnostic category separately (table 3). Results from the within person analyses showed that varenicline was associated with increased hazards for anxiety (hazard ratio 1.27, 1.06 to 1.51) and mood (1.28, 1.07 to 1.52) conditions. However, associations were not significant for psychoses.

**Table 3 tbl3:** Associations between varenicline and incidence of certain psychiatric conditions stratified by pre-existing illness (within person models*)

Psychiatric condition	No of events	Hazard ratio (95% CI)		
		All people	People with pre-existing psychiatric disorders	People without pre-existing psychiatric disorders
Anxiety conditions	3128	1.27 (1.06 to 1.51)	1.23 (1.01 to 1.51)	1.41 (0.99 to 2.00)
Mood conditions	3166	1.28 (1.07 to 1.52)	1.31 (1.06 to 1.63)	1.17 (0.86 to 1.60)
Psychoses	1129	0.94 (0.73 to 1.20)	0.90 (0.70 to 1.16)	3.52 (0.81 to 15.27)

*Within person models compare rate of adverse events when person is prescribed varenicline with rate when same person is not prescribed varenicline.

As we had used a conservative measure of incidence of new psychiatric conditions (that is, excluding all follow-ups and referrals) in all the above analyses, we did sensitivity analyses including emergency visits as well as follow-ups and referrals. Results from these sensitivity analyses showed similar conditions as being significantly associated with varenicline, but with lower effect sizes: increased hazards for anxiety (hazard ratio 1.08, 1.00 to 1.16) and mood conditions (1.09, 1.03 to 1.16) but not for psychoses (1.07, 0.96 to 1.19). To test for confounding by pre-existing psychiatric disorders, we then restricted analyses to people with pre-existing psychiatric disorders (n=493 927) and to those without prior psychiatric disorders (n=7 423 509). Results from the within person analyses showed increased hazards of mood and anxiety conditions only for people with pre-existing psychiatric disorders (table 3).

To test for potential confounding by nicotine withdrawal syndrome, a time varying factor, we did a between person Cox proportional hazards regression that included only people treated with either varenicline or bupropion during follow-up (table 4). Results showed that people treated with varenicline had significantly decreased hazards for mood conditions (hazard ratio 0.63, 0.55 to 0.74) but not for anxiety conditions (0.87, 0.75 to 1.00) compared with those treated with bupropion.

**Table 4 tbl4:** Sensitivity analyses: associations between varenicline and mood and anxiety conditions in people prescribed varenicline compared with those prescribed bupropion*

Condition	No of events in varenicline cohort/No of events in bupropion cohort	Hazard ratio (95% CI): between person, adjusted for sex and age
Anxiety conditions	2517/7590	0.87 (0.75 to 1.00)
Mood conditions	2442/11 653	0.63 (0.55 to 0.74)

*Using between person Cox proportional hazards regression models.

## Discussion

In a large population based cohort of nearly eight million people, of whom 69 757 were treated with varenicline between 2006 and 2009, we investigated associations with suicidal behaviours, criminal offending, psychiatric disorders, transport accidents, and traffic related offences. In between person analyses, adjusted for age and sex, we found that people taking varenicline had increased hazards of the adverse events investigated. However, when we compared periods of treatment with periods of non-treatment within the same person to control for confounding by indication, our principal analytical approach, we found no associations with suicidal behaviour, suspected and convicted criminal offending, transport accidents, or suspected and convicted traffic offences. In addition, the hazard for incidence of new psychiatric conditions was substantially attenuated (from >2 in the between person analyses to 1.2 in the within person analyses), although the risk increase was limited to people with pre-existing psychiatric conditions.

### Strengths and limitations of study

This study improved on previous observational studies through the use of a within person design that adjusts for both residual confounders and confounding by indication.^44^ ^45^ ^46^ ^47^ The study had several strengths, including a large population based cohort with longitudinal data covering several outcomes. Furthermore, information on treatment was complete, as each prescription of varenicline that is collected at pharmacies is registered in the Prescribed Drug Register. Our results suggest that previously reported associations between varenicline and criminal offending and suicidal behaviour are not causal.^9^ ^16^ ^17^ ^18^ Tobacco smokers are more likely to be aggressive and impulsive and have higher rates of suicidal behaviour,^48^ ^49^ ^50^ ^51^ so previous associations are likely to have been confounded by unmeasured factors. This underscores the point that post-marketing surveillance reports are subject to over-reporting and confounding by indication,^29^ ^52^ as well as the need to triangulate data on adverse effects of treatment by using different designs.

Our study is the first, to our knowledge, to examine associations with transport accidents and traffic offences. Previously, transport accidents have been reported as a “strong signal” in post-marketing surveillance events reported to the Food and Drug Administration.^53^ We found no suggestion of a causal association between varenicline and transport accidents and traffic offences in the within person analyses. Thus, the signal identified in post-marketing surveillance data may reflect the overall higher rates of traffic accidents among smokers.^54^ ^55^

Our negative findings are mostly in line with data from randomised controlled trials.^20^ ^21^ ^22^ ^23^ ^28^ ^32^ Our findings have extended those of randomised controlled trials by examining associations in a large cohort sufficiently powered to detect rare events,^33^ ^34^ ^35^ ^36^ ^37^ by studying a wide range of adverse outcomes, and by separately examining people with pre-existing psychiatric diagnoses. The one inconsistency with previous randomised controlled trials is that we found small but statistically significant associations with the incidence of new psychiatric conditions. When explored further, we found no clear association for psychoses, suggesting that previous reported cases of varenicline induced psychoses were not causal.^11^ ^12^ ^13^ ^14^ ^15^ However, the risk remained for anxiety (hazard ratio 1.27, 1.06 to 1.51) and mood (1.28, 1.07 to 1.52) conditions. When we stratified on psychiatric history, associations remained only for people with a history of psychiatric conditions. It has been argued that varenicline is highly selective for α4 β2 nicotinic receptors and at therapeutic concentrations does not bind to other neurotransmitter receptors and transporters, including those implicated in mental health problems.^56^ ^57^ The within person analyses, however, did not take time varying confounding factors into account—that is, factors that were associated with both the start of varenicline treatment and the outcome. The increased risk of mood and anxiety conditions during varenicline treatment in this group could thus be caused by time varying factors other than varenicline; the start of varenicline treatment could indicate non-adherence to other drugs, which may lead to increased incidence of new psychiatric conditions. The fact that randomised controlled trials have not shown an increase in depressive symptoms among people who are on stable treatment for their depression would support this view.^35^ An alternative explanation is that nicotine withdrawal is a time varying confounder. When deprived of nicotine, nicotine dependent people can have withdrawal symptoms that include depression and anxiety, as nicotine includes psychoactive compounds that mimic the antidepressant effects of monoamine oxidase inhibitors.^56^ ^58^ To test for potential confounding by nicotine withdrawal symptoms, we compared people treated with varenicline with those treated with bupropion. Our results showed that people treated with varenicline had a lower risk of mood conditions and showed no difference in risk for anxiety conditions compared with those treated with bupropion. The similar or higher risks of psychiatric conditions in another cohort of smokers would support the view that the increased risk found for varenicline in the within person analyses could be confounded by smoking cessation itself. The risk of mood and anxiety conditions among varenicline users reported here should thus be regarded with caution and needs to be confirmed in further studies.

In addition to the lack of information on time varying covariates, other limitations include the use of official registers, which underestimate true rates of most outcomes; only outcomes serious enough to warrant emergency visits or hospital admission (for psychiatric conditions, transport accidents, or suicidal behaviours) or detection by the police (for crime outcomes), would end up in the registers. On the other hand, register based outcomes are more comparable across countries than self reports and represent important public health concerns. Furthermore, although our data on collected prescriptions is complete, it is unable to account for lack of or variations in adherence. This problem is parallel to non-adherence in randomised controlled trials, and our within person estimate is comparable to the intention to treat analysis used in randomised controlled trials. Finally, our study was conducted in Sweden, a country with a relatively low prevalence of daily smokers in international comparisons^59^; the prevalence of adult daily smokers is 14% compared with an average of 23% in the European Union.^60^ Differences in smoking rates, as well as in varenicline prescriptions, may thus affect the generalisability of results. Nevertheless, our findings in relation to suicidal outcomes are in line with cohort studies using different designs from the United Kingdom and Denmark.^24^ ^26^ ^27^

### Conclusions and implications for further research

In summary, our results provide no evidence for a causal association between varenicline and the incidence of criminal offending, suicidal behaviour, transport accidents, traffic offences, and psychoses. However, an increased risk of mood and anxiety conditions during periods of varenicline treatment was found in people with pre-existing psychiatric disorders, which needs to be confirmed using other study designs.

**What is already known on this topic**

Varenicline is widely prescribed to treat nicotine dependence, but reports of suicidality, depression, psychoses, and violence have emerged, leading to warnings being issued by regulatory agencies
Varenicline use has also been restricted or prohibited for pilots, air traffic controllers, truck and bus drivers, and certain military personnel owing to reports of traffic accidents
However, case reports and safety results from post-marketing surveillance studies of varenicline are not consistent with observational data and results of randomised controlled trials

**What this study adds**

A within person design was used to minimise selection effects and adjust for unknown confounders and confounding by indication in a large population based cohort
No evidence was found for a causal association between varenicline and criminal offending, suicidal behaviour, transport accidents, traffic offences, or psychoses
However, an increased risk of mood and anxiety conditions was found in people with pre-existing psychiatric disorders, which needs to be confirmed using other study designs

## Notes

Contributors: YM was involved in the conception of the study, analysis and interpretation of the data, and writing the manuscript. SF was involved in the conception of the study, interpretation of the data, and writing the manuscript. JZ was involved in the study design and in analysis and interpretation of the data. PL and CHG were involved in study conception and interpretation of the data. All authors were involved in revising the article critically for important intellectual content and final approval of the version to be published. SF is the guarantor.

Ethical approval: This study was approved by the ethical committee of Karolinska Institutet (2005/4:5).

Transparency declaration: The lead author (the manuscript’s guarantor) affirms that this manuscript is an honest, accurate, and transparent account of the study being reported; that no important aspects of the study have been omitted; and that any discrepancies from the study as planned (and, if relevant, registered) have been explained.

Data sharing: No additional data available.

## Notes

Cite this as: *BMJ* 2015;350:h2388

## References

[1] Shafey O, Dolwick S, Guindon GE, eds. Tobacco control country profiles. 2nd ed. American Cancer Society, 2003 (available at www.who.int/tobacco/global_data/country_profiles/en/).

[2] Lim SS, Vos T, Flaxman AD, et al. A comparative risk assessment of burden of disease and injury attributable to 67 risk factors and risk factor clusters in 21 regions, 1990-2010: a systematic analysis for the Global Burden of Disease Study 2010. Lancet 2012;380:2224-60. 23245609 10.1016/S0140-6736(12)61766-8 PMC4156511

[3] World Health Organization. Global health risks: mortality and burden of disease attributable to selected major risks. WHO, 2009.

[4] Cahill K, Stevens S, Perera R, Lancaster T. Pharmacological interventions for smoking cessation: an overview and network meta-analysis. Cochrane Database Syst Rev 2013;5:CD009329. 23728690 10.1002/14651858.CD009329.pub2 PMC8406789

[5] Food and Drug Administration. FDA drug safety communication: safety review update of Chantix (varenicline) and risk of neuropsychiatric adverse events. 2011. www.fda.gov/Drugs/DrugSafety/ucm276737.htm.

[6] National Institute for Health and Clinical Excellence. NICE implementation uptake report: smoking cessation drugs. NICE, 2010.

[7] Food and Drug Administration. FDA issues public health advisory on Chantix. 2008. www.fda.gov/NewsEvents/Newsroom/PressAnnouncements/2008/ucm116849.htm.

[8] Medicines and Healthcare Products Regulatory Agency (MHRA). Varenicline: possible effects on driving; psychiatric illness. Drug Safety Update 2007;1:12.

[9] Moore TJ, Furberg CD, Glenmullen J, Maltsberger JT, Singh S. Suicidal behavior and depression in smoking cessation treatments. PLoS One 2011;6:e27016. 22073240 10.1371/journal.pone.0027016 PMC3206890

[10] Kuehn BM. Varenicline gets stronger warnings about psychiatric problems, vehicle crashes. JAMA 2009;302:834. 19706852 10.1001/jama.2009.1153

[11] Annagur BB, Bez Y. Varenicline-induced psychotic depressive episode in a patient with bipolar disorder. Ther Adv Psychopharmacol 2012;2:35-7. 23983954 10.1177/2045125311430111 PMC3736926

[12] Cinembre B, Akdag ST, Metin O, Doganavsargil O. Varenicline-induced psychosis. CNS Spectr 2010;15:469-72. 20676055 10.1017/s1092852900000407

[13] DiPaula BA, Thomas MD. Worsening psychosis induced by varenicline in a hospitalized psychiatric patient. Pharmacotherapy 2009;29:852-7. 19558259 10.1592/phco.29.7.852

[14] Forcen FE, Martinez FL, Moya AM. Varenicline precipitating psychosis in a patient with no previous psychiatric history: a case report of a Spanish patient who was later diagnosed with paranoid personality disorder. Clin Schizophr Relat Psychoses 2012;5:221-3. 22182460

[15] Gupta A, Bastiampillai T, Adams M, Nelson, A, Nance, M. Varenicline induced psychosis in schizophrenia. Aust N Z J Psychiatry 2012;46:1009. 22495951 10.1177/0004867412444993

[16] Aagaard L, Hansen EH. Adverse drug reactions reported by consumers for nervous system medications in Europe 2007 to 2011. BMC Pharmacol Toxicol 2013;14:30. 23763896 10.1186/2050-6511-14-30 PMC3685574

[17] Harrison-Woolrych M, Ashton J. Psychiatric adverse events associated with varenicline: an intensive postmarketing prospective cohort study in New Zealand. Drug Saf 2011;34:763-72. 21830838 10.2165/11594450-000000000-00000

[18] Moore TJ, Glenmullen J, Furberg CD. Thoughts and acts of aggression/violence toward others reported in association with varenicline. Ann Pharmacother 2010;44:1389-94. 20647416 10.1345/aph.1P172

[19] Meyer, TE, Taylor LG, Xie S, et al. Neuropsychiatric events in varenicline and nicotine replacement patch users in the military health system. Addiction 2013;108:203-10. 22812921 10.1111/j.1360-0443.2012.04024.x

[20] Cincipirini PM, Robinson JD, Karam-Hage M, Minnix JA, Lam C, Versace F. Effects of varenicline and bupropion sustained-release use plus intensive smoking cessation counseling on prolonged abstinence from smoking and on depression, negative affect, and other symptoms of nicotine withdrawal. JAMA Psychiatry 2013;70:522-33. 23536105 10.1001/jamapsychiatry.2013.678 PMC4128024

[21] Foulds J, Russ C, Yu C-R, et al. Effect of varenicline on individual nicotine withdrawal symptoms: a combined analysis of eight randomized, placebo-controlled trials. Nicotine Tob Res 2013;15:1849-57. 23694782 10.1093/ntr/ntt066

[22] Garza D, Murphy M, Tseng L-J, Riordan HJ, Chatterjee A. A double-blind randomized placebo-controlled pilot study of neuropsychiatric adverse events in abstinent smokers treated with varenicline or placebo. Biol Psychiatry 2011;69:1075-82. 21295286 10.1016/j.biopsych.2010.12.005

[23] Gibbons RD, Mann JJ. Varenicline, smoking cessation, and neuropsychiatric adverse events. Am J Psychiatry 2013;170:1460-7. 24030388 10.1176/appi.ajp.2013.12121599 PMC4238282

[24] Gunnell D, Irvine D, Wise L, Davies C, Martin RM. Varenicline and suicidal behaviour: a cohort study based on data from the General Practice Research Database. BMJ 2009;339:b3805. 19797344 10.1136/bmj.b3805 PMC2755726

[25] Nahvi S, Wu B, Richter KP, Bernstein SL, Arnsten JH. Low incidence of adverse events following varenicline initiation among opioid dependent smokers with comorbid psychiatric illness. Drug Alcohol Depend 2013;132:47-52. 23332438 10.1016/j.drugalcdep.2012.12.026 PMC3762456

[26] Pasternak B, Svanström H, Hviid A. Use of varenicline versus bupropion and risk of psychiatric adverse events. Addiction 2013;108:1336-43. 23445269 10.1111/add.12165

[27] Thomas KH, Martin RM, Davies NM, Metcalfe C, Windmeijer F, Gunnell D. Smoking cessation treatment and risk of depression, suicide, and self harm in the Clinical Practice Research Datalink: prospective cohort study. BMJ 2013;347:f5704. 24124105 10.1136/bmj.f5704 PMC3805476

[28] Tonstad S, Davies S, Flammer M, Russ C, Hughes J. Psychiatric adverse events in randomized, double-blind, placebo-controlled clinical trials of varenicline: a pooled analysis. Drug Saf 2010;33:289-301. 20297861 10.2165/11319180-000000000-00000

[29] Gibbons RD, Amatya AK, Brown CH, et al. Post-approval drug safety surveillance. Annu Rev Public Health 2010;31:419-37. 20070192 10.1146/annurev.publhealth.012809.103649 PMC2841706

[30] Kalman D, Baker Morissette S, George TP. Co-morbidity of smoking in patients with psychiatric and substance use disorders. Am J Addict 2005;14:106-23. 16019961 10.1080/10550490590924728 PMC1199553

[31] Kuehn BM. New reports examine psychiatric risks of varenicline for smoking cessation. JAMA 2012:307:129-30. 10.1001/jama.2011.1924 22235073

[32] Thomas KH, Martin RM, Knipe DW, Higgins JPT, Gunnell D. Risk of neuropsychiatric adverse events associated with varenicline: systematic review and meta-analysis. BMJ 2015;350:h1109. 25767129 10.1136/bmj.h1109 PMC4357491

[33] Shim JC, Jung DU, Jung SS, et al. Adjunctive varenicline treatment with antipsychotic medications for cognitive impairments in people with schizophrenia: a randomized double-blind placebo-controlled trial. Neuropsychopharmacology 2012:37:660-8. 10.1038/npp.2011.238 PMC3260973 22048460

[34] Chengappa KN, Perkins KA, Brar JS, et al. Varenicline for smoking cessation in bipolar disorder: a randomized, double-blind, placebo-controlled study. J Clin Psychiatry 2014;75:765-72. 25006684 10.4088/JCP.13m08756

[35] Anthenelli RM, Morris C, Ramey TS, et al. Effects of varenicline on smoking cessation in adults with stably treated current or past major depression: a randomized trial. Ann Intern Med 2013;159:390-400. 24042367 10.7326/0003-4819-159-6-201309170-00005

[36] Evins AE, Cather C, Pratt SA, et al. Maintenance treatment with varenicline for smoking cessation in patients with schizophrenia and bipolar disorder: a randomized clinical trial. JAMA 2014;311:145-54. 24399553 10.1001/jama.2013.285113 PMC4124884

[37] Food and Drug Administration. FDA Drug Safety Communication: Safety review update of Chantix (varenicline) and risk of neuropsychiatric adverse events. 2011. www.fda.gov/drugs/drugsafety/ucm276737.htm.

[38] Wettermark B, Hammar N, MichaelFored C, et al. The new Swedish Prescribed Drug Register—opportunities for pharmacoepidemiological research and experience from the first six months. Pharmacoepidemiol Drug Saf 2007;16:726-35 16897791 10.1002/pds.1294

[39] Hays JT, Ebbert JO. Varenicline for tobacco dependence. N Engl J Med 2008;359:2018-24. 18987369 10.1056/NEJMct0800146 PMC2959114

[40] National Council for Crime Prevention. Kriminalstatistik 2010 [Criminal statistics 2010]. National Council for Crime Prevention, 2010.

[41] Fazel S, Grann M. The population impact of severe mental illness on violent crime. Am J Psychiatry 2006;163:1397-403. 16877653 10.1176/ajp.2006.163.8.1397

[42] Ludvigsson J, Andersson E, Ekbom A, et al. External review and validation of the Swedish national inpatient register. BMC Public Health 2011;11:450. 21658213 10.1186/1471-2458-11-450 PMC3142234

[43] Johansson LA, Westerling R. Comparing Swedish hospital discharge records with death certificates: implications for mortality statistics. Int J Epidemiol 2000;29:495-502. 10869322

[44] Chang Z, Lichtenstein P, D’Onofrio BM, Sjölander A, Larsson H. Serious transport accidents in adults with attention-deficit/hyperactivity disorder and the effect of medication: a population-based study. JAMA Psychiatry 2014;71:319-25. 24477798 10.1001/jamapsychiatry.2013.4174 PMC3949159

[45] Chen Q, Sjolander A, Runeson B, D’Onofrio BM, Lichtenstein P, Larsson H. Drug treatment for attention-deficit/hyperactivity disorder and suicidal behaviour: register based study. BMJ 2014;348:g3769. 24942388 10.1136/bmj.g3769 PMC4062356

[46] Fazel S, Zetterqvist J, Larsson H, Långström N, Lichtenstein N. Antipsychotics, mood stabilisers, and risk of violent crime. Lancet 2014;384:1206-14. 24816046 10.1016/S0140-6736(14)60379-2 PMC4165625

[47] Lichtenstein P, Halldner L, Zetterqvist J, et al. Medication for attention deficit- hyperactivity disorder and criminality. N Engl J Med 2012;367:2006-14. 23171097 10.1056/NEJMoa1203241 PMC3664186

[48] Baek J-H, Eisner L-R, Nierenberg AA. Smoking and suicidality in subjects with major depressive disorder: results from the National Epidemiologic Survey on Alcohol and Related Conditions (NESARC). J Affect Disord 2013;150:1158-66. 23827531 10.1016/j.jad.2013.05.082

[49] Dakwar E, Popii M, Coccaro EF. Lifetime history of cigarette smoking associated with aggression and impulsivity in both healthy and personality disordered volunteers. J Pers Disord 2011;25:645-55. 22023301 10.1521/pedi.2011.25.5.645 PMC3594993

[50] Holma IA, Holma KM, Melartin TK, Ketokivi M, Isometsa ET. Depression and smoking: a 5-year prospective study of patients with major depressive disorder. Depress Anxiety 2013;30:580-8. 23606190 10.1002/da.22108

[51] Schneider B, Lukaschek K, Baumert J, Meisiner M, Erazo N, Ladwig K-H. Living alone, obesity, and smoking increase risk for suicide independently of depressive mood findings from the population-based MONICA/KORA Augsburg cohort study. J Affect Disord 2014;152-154:416-21. 10.1016/j.jad.2013.10.007 24238953

[52] Mayet A, Nivoix P, Haus-Cheymol R, et al. Increase in reported adverse events following seasonal influenza vaccination among the French armed forces, 2008-2009: possible role of stimulated reporting and background cases of influenza-like infection. Public Health 2012;126:70-6. 22137096 10.1016/j.puhe.2011.09.025

[53] Institute of Safe Medicine Practices. Strong safety signal seen for new varenicline risks. 2008. www.ismp.org/docs/vareniclinestudy.asp.

[54] Yiengprugsawan V, Berecki-Gisolf J, Bain C, McClure R, Seubsman S-A, Sleigh AC. Predictors of injury mortality: findings from a large national cohort in Thailand. BMJ Open 2014;4:e004668. 10.1136/bmjopen-2013-004668 PMC4054638 24902726

[55] Wen CP, Tsai SP, Cheng TY, Chan HT, Chung WS, Chen CJ. Excess injury mortality among smokers: a neglected tobacco hazard. Tob Control 2005;14(suppl 1):i28-32. 15923444 10.1136/tc.2003.005629 PMC1766175

[56] Hughes JR. Smoking and suicide: a brief overview. Drug Alcohol Depend 2008;98:169-78. 18676099 10.1016/j.drugalcdep.2008.06.003 PMC2585177

[57] Kaur K, Kaushal S, Chopra SC. Varenicline for smoking cessation: a review of the literature. Curr Ther Res Clin Exp 2009;70:35-54. 24692831 10.1016/j.curtheres.2009.02.004 PMC3969980

[58] Ahmed AI, Ali AN, Kramers C, Härmark LV, Burger DM, Verhoeven WM. Neuropsychiatric adverse events of varenicline: a systematic review of published reports. J Clin Psychopharmacol 2013;33:55-62. 23277249 10.1097/JCP.0b013e31827c0117

[59] Ng M, Freeman MK, Fleming TD, et al. Smoking prevalence and cigarette consumption in 187 countries, 1980-2012. JAMA 2014;311:183-92. 24399557 10.1001/jama.2013.284692

[60] Organisation for Economic Co-operation and Development. Health at a glance: Europe 2012. OECD Publishing, 2012 (available at http://dx.doi.org/10.1787/9789264183896-en).

